# Fiber orientation‐dependent T_1_ angular features in human white matter at 1.5 T, 3 T, and 7 T

**DOI:** 10.1002/mrm.70009

**Published:** 2025-07-25

**Authors:** Risto A. Kauppinen, Ekaterina Paasonen, Jeromy Thotland, Mervi Könönen, Pramod Pisharady, Christophe Lenglet, Juhana M. Hakumäki, Olli H. J. Gröhn, Michael Garwood

**Affiliations:** ^1^ Department of Electric, Electronic and Mechanical Engineering University of Bristol Bristol UK; ^2^ A.I.Virtanen Institute, University of Eastern Finland Kuopio Finland; ^3^ Kuopio University Hospital Neurocenter Kuopio Finland; ^4^ Center for Magnetic Resonance Research, University of Minnesota Minneapolis Minnesota USA; ^5^ Department of Radiology University Hospital of Kuopio Kuopio Finland

**Keywords:** fiber orientation, magnetic field strength, T_1_ relaxation, white matter

## Abstract

**Purpose:**

White matter (WM) microstructure influences T_1_ in vivo. Here, T_1_ angular features were studied in human WM in vivo in quantitative terms at 1.5 T, 3 T and 7 T.

**Methods:**

MP2RAGE MRI was used to compute absolute T_1_ images of the brain at three fields. Diffusion MRI images were acquired using the manufacturer's diffusion MRI (dMRI) protocol at 1.5 T and Human Connectome Project protocols at 3 T and 7 T to compute WM microstructural DTI indices. Axonal fiber‐to‐field dependency of T_1_ relaxation was determined in WM and the quantitative characteristics of the angular features were measured in absolute terms at all three fields.

**Results:**

Two angular features in T_1_ relaxation were present in WM with fractional anisotropy >0.5 at all three fields showing the characteristics: (1) increasing T_1_ relaxation from parallel to perpendicular orientations of axonal fibers and (2) a broad long T_1_ hump centered around 40° orientation in respect to the field. The former feature amounted to 4.5% to 4.9% of average T_1_ at three fields, the latter was 4.2% to 3.4% of average T_1_ at 1.5 T and 3 T, but only to 1.8% at 7 T. The angular plots of signals in TI images indicated much shorter T_1_ in the proton species underpinning the 40° feature at 1.5 T than at 7 T, which may underpin the quantitative difference of the 40° hump between the fields.

**Conclusions:**

T_1_ relaxation anisotropy is an inherent WM MRI contrast that is detectable at all MR fields most commonly used in human neuroimaging, and it should be considered in the evaluations of quantitative T_1_ MRI.

## 1 INTRODUCTION

The central role of T_1_ contrast in a number of basic and clinical neuroscientific applications, including volumetry of brain structures, cortical thicknesses, and shape analyses,^1^, ^2^, ^3^ has motivated extensive research on physics of longitudinal relaxation in the two main brain tissue types, the gray matter (GM) and white matter (WM).^4^, ^5^, ^6^, ^7^ It is commonly accepted that magnetization exchange between bulk water and immobilized macromolecular protons is the key pathway influencing T_1_ relaxation in both GM and WM.^8^ Magnetization exchange, often referred to as the magnetization transfer (MT), has been adequately modeled in the context of the so‐called binary spin bath (BSB) system consisting of immobilized macromolecular protons and mobile bulk water hydrogens both with inherent relaxivities.^8^ MT involves both the through‐space dipole interaction and chemical exchange of hydrogens between the two pools. As stipulated by the BSB model, the multi‐exponential characteristics of T_1_ relaxation in neural tissue have been observed experimentally.^9^, ^10^ According to the BSB model the short T_1_ species corresponds to the motionally restricted protons in membranes and axonal myelinated structures. In addition, a pool of protons with both short T_1_ (T_1_ ˜150 ms at 3 T) and short T_2_* (T_2_* ˜29 ms at 3 T) has been attributed to the myelin‐associated water^9^ and another to those water protons undergoing MT with immobile species.^11^ The fraction of the myelin‐associated water increases in the T_1_ relaxogram from 3 T to 7 T while its T_1_ stays unchanged.^9^ Therefore, at least three distinct proton pools may contribute to the short T_1_ component, all these proton pools are likely to underpin to the multi‐exponential behavior of T_1_ in WM. Nevertheless, the recent evidence points to the motionally restricted species in membranes and myelinated axons with inherent short correlation times causing the B_0_‐depndency of T_1_.^12^, ^13^ In fact, the B_0_‐dependency of T_1_ in the brain has been attributed to be because of the inherent nature of magnetic interaction between the motionally restricted bound protons in hydrophilic lipid heads with bulk protons.^13^

Evidence is accumulating that several WM microstructure features modulate T_1_ relaxation and MT,^14^, ^15^ including axon diameter,^16^, ^17^ fiber configuration^18^, ^19^ and orientation of axon fibers with respect to the main magnetic field.^20^, ^21^, ^22^, ^23^ Although the effects of microstructural factors on overall T_1_ are weak relative to those of MT and the water‐to‐macromolecule ratio, they render T_1_ to an MRI contrast with a potential for imaging unprecedented properties of WM microstructure and to complement those obtained by other MRI methods. In particular, this concerns orientation dependency, as the effect of WM microstructure to T_1_ takes place at a nanometer scale.^23^, ^24^ The T_1_ angular dependency in WM has been firmly linked to the relaxation anisotropy by experimental evidence from ex vivo brain preparations rotated in the magnetic field.^25^ Using inversion recovery (IR)‐based pulse sequences at 9.4 T to measure T_1_ in a rat splenium ex vivo it was reported that T_1_ relaxation increases from parallel to perpendicular orientation and that a long T_1_ feature is present when axonal fibers are rotated to 30° to 50° angle relative to B_0_.^25^ In an ex vivo spinal cord preparation, the longest T_1_ was found at 40° rotation, the shortest at 90° angle, and an intermediate value at 0° as measured either by inversion recovery turbo spin echo (IR‐TSE) or MP2RAGE at 3 T.^23^ Interestingly, the T_1_ angular patterns observed in ex vivo WM preparations closely resembled those reported in human WM in vivo as measured by MP2RAGE MRI.^25^ Instead, angular patterns obtained using variable flip angle (VFA) MRI showed only increasing T_1_ relaxation from parallel to perpendicular orientations in WM in vivo, but not the 40° feature.^20^ Recently devised physical models provide a solid footing for T_1_ anisotropy as a MR contrast originating from hydrogens of macromolecules present in anisotropic physico‐chemical environments and their magnetic interactions with bulk water protons.^13^, ^14^, ^26^, ^27^

Given the field dependency of T_1_, it is important to examine the quantitative behavior of angularly dependent longitudinal relaxation in vivo at different B_0_s. The current study was undertaken to measure T_1_ in WM using IR‐based MP2RAGE MRI with consistent pulsing conditions and to examine the quantitative characteristics of T_1_ angular features at magnetic field strengths that are relevant to neuroimaging both in clinical and investigational neuroscience. We have quantified T_1_ angular features with the objective to evaluate the effects of B_0_ on the anisotropy characteristics. Furthermore, by using consistent MRI acquisitions at all fields, we expect that the data may aid in unraveling physical mechanisms underpinning the T_1_ relaxation anisotropy in vivo.

## 2 METHODS

### 2.1 Human subjects

The study protocols received ethical approvals from North Savo Research Ethics Board (Kuopio, Finland) and the University of Minnesota institutional review board (Minneapolis, MN, USA). Informed consent was obtained before accrual from each volunteer. Eight healthy volunteers (mean age, 33 years; range, 28–37 years, six females) consented to participate the study for 1.5 T (exact B_0_ 1.495 T, Larmor frequency 63.661 MHz) scans in the University Hospital of Kuopio. Six healthy volunteers (age range, 23–32 years, two females) were accrued in Minneapolis and they were scanned both at 3 T (exact B_0_ 2.89 T, Larmor frequency 123.174 MHz) and 7 T (exact B_0_ 6.98 T, Larmor frequency 297.208 MHz) within a period of 6 to 9 months.

### 2.2 MRI

A Siemens 1.5 T MAGNETOM Sola system with a 20 channel head coil was used at the University Hospital of Kuopio, whereas a Siemens MAGNETOM Prisma 3 T system with a 32 channel head coil and a Siemens MAGNETOM 7 T AS scanner with a Nova Medical 1 transmit/32 receive head coil were used at Center for Magnetic Resonance Research, University of Minnesota. MP2RAGE sequences were used at all field strengths to acquire TI series for T_1_ mapping (Table S1 for acquisition parameters). Adiabatic inversion pulses in the MP2RAGE sequence had comparable durations at each field as follows: 10 199 μs (1.5 T), 10 240 μs (3 T), and 10 800 μs (7 T). Alpha pulses of 4° in the readout modules were used for consistent measurement conditions (Table S1). To evaluate the possible influence of on‐resonance saturation by the hard read pulses, 8° read flip MP2RAGE images were also collected in the same imaging session at 3 T. Diffusion MRI (dMRI) images were acquired using the single‐shot EPI pulse sequence according to the manufacture's protocol (EPI_2D DIFF) at 1.5 T, the Human Connectome Project (HCP) Lifespan Protocol^28^ at 3 T and the HCP Young Adult Protocol^29^ at 7 T with the parameters shown in Table S1. All dMRI scans were acquired without angulation at scanner coordinates. B_1_ maps were also acquired at 3 T and 7 T at resolution of 4 × 4 × 8 mm^3^ using the manufacturer's routine.

### 2.3 Image processing

dMRI scans were corrected for distortions because of eddy currents, susceptibility‐induced off‐resonance artifacts and subject motion using TOPUP and EDDY in FMRIB Software Library (FSL).^30^, ^31^ Denoising of 1.5 T diffusion images was performed before further processing using the noise reduction with distribution corrected principal component analysis (NORDIC) method.^32^ A DTI model was subsequently fitted to the corrected data using DTIFIT in FSL,^33^ to compute the DTI indices (fractional anisotropy [FA], mean diffusivity, V_1_, V_2_, and V_3_) using b = 0 s/mm^2^ and b = 750 s/mm^2^ images at 1.5 T, b = 0 s/mm^2^ and b = 1500 s/mm^2^ images at 3 T and b = 0 and b = 1000 s/mm^2^ at 7 T. The general consensus is that the optimal b‐value lies within 700 and 1500 s/mm^2^, with 1000 s/mm^2^ being the most commonly used value.^34^ We used the principal eigenvector images (V_1_) from the DTI model for the estimate of fiber orientations. The ability of the DTI model to resolve fiber orientations in voxels with complex fiber configurations, such as in crossing fiber configurations, is known to be problematic.^35^, ^36^ To minimize this issue, the FA cutoff was set at 0.5, above which the crossing fiber issue is less prominent.^37^ The FA cutoff at 0.5 has been used in recent studies on orientation dependency of relaxation times in WM in vivo.^20^, ^21^, ^38^ We also conducted analyses by increasing the FA threshold to FA >0.65 (Figure S1). These analyses provided virtually identical T_1_ angular plots as seen in WM with FA cutoff set at 0.5, yet with a slight offset in T_1_ and R_1_ values in the two FA ranges (Figure S1) because of the difference in FAs.^19^ We have previously conducted analyses on T_1_ angular features in WM in vivo using the primary fiber orientations from the ball‐and‐stick crossing fiber model, which also resolved multiple fiber orientations and fiber‐to‐field angles.^19^ For the purpose of the current study, the fiber‐to‐field (θ_FB_) maps were computed from the principal direction of diffusion using the principal eigenvector V_1_ images and direction of B_0_ as previously described.^21^

The SNR was computed from the diffusion images using the procedure described in Descoteaux et al.^39^ and implemented in diffusion imaging in Python documentation.^40^ A mask encompassing the midsagittal portion of the entire length of the corpus callosum (CC) in anterior–posterior direction was used to measure SNR in images (Table S2). It is evident from Table S2 that even in the worst case (*x*‐gradient direction, as CC fibers are positioned close to *x* direction receiving the most attenuated diffusion signal) the SNRs were well above the noise floor bias at all fields.^41^

Signal intensities in TI images of MP2RAGE scans depend on T_1_, readout flip angle, TR, and other acquisition parameters (Table S1), hence, T_1_ from a set of TI images can be calculated using Bloch equations.^42^ The TI images from the TI pairs were first registered to the longest TI images. T_1_ maps from all six TI images were computed assuming a single ensemble of longitudinal magnetization in each voxel and the signal intensities in TI images were fitted into a mono‐exponential model in MATLAB (MathWorks, Natick, MA, USA) as previously described.^21^ The sign problem in the magnitude signals was solved by performing several fits in which points at or close to the minimum signal intensity and all those to their left (i.e., shorter TI) were allowed to change sign, the best fit being retained, using a direct Nelder–Mead simplex search. T_1_ maps were also computed from the TI images with four longest delay times by fitting the magnitude data into a mono‐exponential with a least‐squares weighting. Magnitude signal intensities in the four TI images were assumed to be positive. These T_1_ maps are referred to as long‐TI T_1_ maps.

The FA maps were registered to the R_1_ (R_1_ = 1/ T_1_) images with FLIRT in FSL,^43^ and V_1_ images were then registered on the R_1_ maps using the transformation matrix from FA image registrations. 1D and 2D plots of T_1_ and individual TI image signal intensities as a function of $\theta_{\mathrm{FB}}$ and FA were computed in MATLAB as previously described.^21^ WM masks were obtained from R_1_ maps using BET2 in FSL. The masks were visually inspected against FA images and refined by eroding the outermost voxels as required. Student's paired and unpaired t‐test was used for statistical testing as indicated.

## 3 RESULTS

**FIGURE 1:**
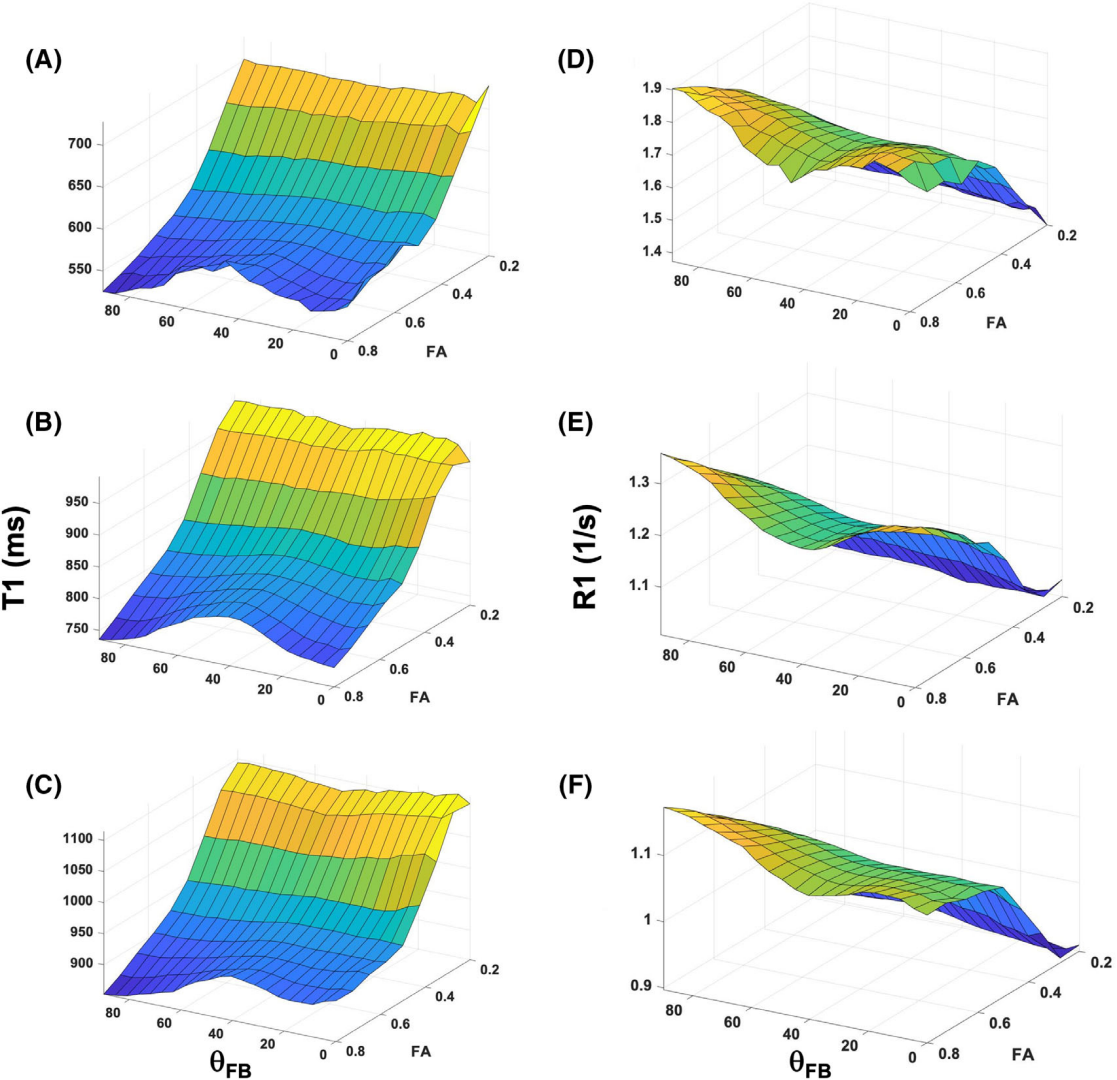
Two dimensional T_1_ versus fiber‐to‐field angle and R_1_ versus fiber‐to‐field‐angle plots as function of FA. (A,D) From 1.5 T, (B,E) from 3 T, and (C,F) from 7 T. White matter (WM) volumes with fractional anisotropy (FA) from 0.2 to 0.8 are included. Data at 1.5 T are from eight and at 3 T and 7 T from six volunteers.

**FIGURE 2:**
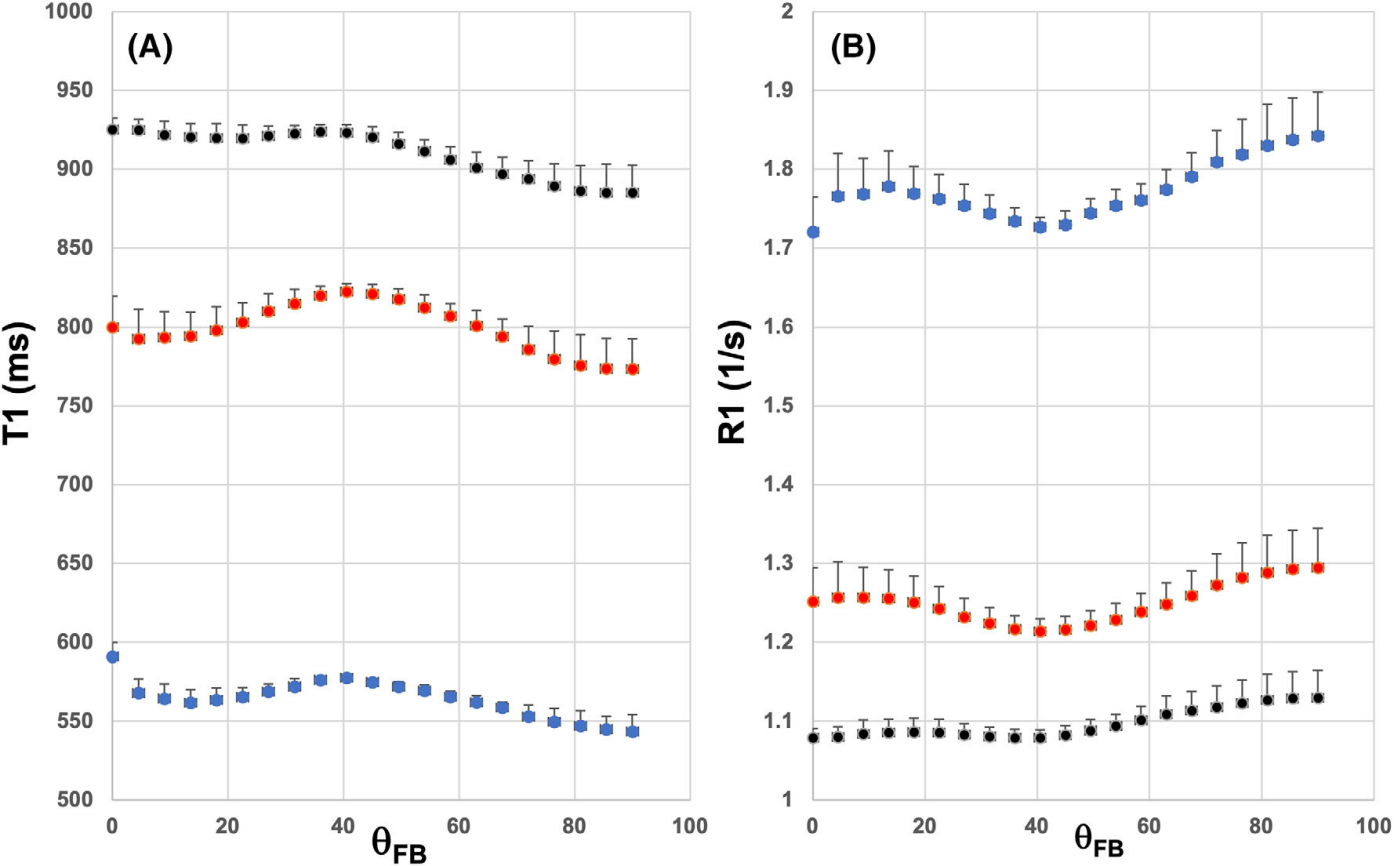
T_1_ and R_1_ versus fiber‐to‐field‐angle plots from human white matter (WM) with high FA. (A) Shows T_1_ (in ms) versus θ_FB_ plots for WM with 1.5 T (blue symbols), 3 T (red symbols), and 7 T (black symbols). (B) Shows the respective R_1_ (in 1/s) versus θ_FB_ plots. Data are from WM with the fractional anisotropy (FA) range from 0.5 to 0.8 at 1.5 T from eight, at 3 T and 7 T from six volunteers. Data are mean ± S.D.

Two dimensional plots of T_1_ (Figure 1A–C) and R_1_ (Figure 1D–F) as a function of FA and θ_FB_ are shown for all three B_0_s. It is evident that a broad hump in T_1_ and a respective broad dip in R_1_ appeared around θ_FB_ of 40° in WM with FA higher ˜0.5 at all fields. Conversely, no consistent angular features are seen in FA <0.4. We more closely studied the angular patterns of both T_1_ and R_1_ in WM with FA >0.5, termed as the high FA WM (with FA values as follows: 0.636 ± 0.041, 0.636 ± 0.042, and 0.639 ± 0.042 at 1.5 T, 3 T, and 7 T, respectively). T_1_ and R_1_ as a function θ_FB_ were plotted in high FA WM (Figure 2). These plots demonstrate that the difference in T_1_ (Figure 2A) and R_1_ (Figure 2B) values between 0° and 90° appeared greater at 1.5 T than at 7 T. Similarly, the size of the broad hump in T_1_ versus θ_FB_ (Figure 2A) and broad dip in R_1_ versus θ_FB_ (Figure 2B) around 40° appeared shallower when moving from 1.5 T to 7 T The absolute values for the two angular features of (1) increasing longitudinal relaxation from 0° to 90° and (2) the 40° centered hump in T_1_ (in ms) and R_1_ (in 1/s) plots are shown in Table 1. The differences in T_1_s in WM where fibers run parallel and perpendicular varied from ˜27 ms at 1.5 T to ˜41 ms at 7 T. The respective difference in R_1_ plots varied from ˜0.082 1/s at 1.5 T to ˜0.050 1/s at 7 T. All the differences between 0° and 90° were ˜4% to 5% of average T_1_ and R_1_ at three B_0_s (Table 1). The broad 40° humps (in ms) varied from ˜24 ms at 1.5 T to ˜16 ms at 7 T, with respective values in R_1_ plots of ˜0.066 1/s and ˜0.019 1/s. The 40° features was significantly smaller at 7 T than those at 1.5 T or 3 T (i.e., ˜1.8% vs ˜4%) (Table 1). The FWHM values of the 40° humps were similar ranging from 27° to 34° (Table 1). The results indicate that the two angular features, the increasing longitudinal relaxation from parallel to perpendicular fibers and the hump at 40°, were quantitatively different at 7 T from those at 1.5 T and 3 T (Table 1).

**TABLE 1:** Quantitative characteristics of T_1_ and R_1_ angular features together with absolute T_1_ and R_1_ data from human WM.

Field	0°–90° (ms)	% of T_1_	0°–90° (1/s) × 10^−3^	% of R_1_	40° feature (ms)	% of T_1_	40° feature (1/s) × 10^−3^	% of R_1_	T_1_ (ms)	R_1_ (1/s)	FWHM (°)
1.5 T	27.4 ± 11.7	4.9 ± 2.1	81.9 ± 15.2	4.6 ± 0.9	23.5 ± 6.1	4.2 ± 1.1	65.9 ± 12.5	3.7 ± 0.6	564.1 ± 26.7	1.774 ± 0.090	34.2 ± 3.2
3 T	36.2 ± 10.1	4.5 ± 1.3	49.1 ± 17.0*	3.7 ± 1.4	27.5 ± 8.5	3.4 ± 1.0	57.4 ± 15.9	4.6 ± 1.3	808.0 ± 2.6	1.238 ± 0.004	34.6 ± 3.6
7 T	41.3 ± 11.1	4.5 ± 1.2	50.3 ± 15.8*	4.6 ± 1.4	16.5 ± 4.1^‡^	1.8 ± 0.4*	19.2 ± 6.8^‡^	1.7 ± 0.6^‡^	911.3 ± 18.4	1.098 ± 0.011	27.0 ± 6.0
1.5 T (2)	31.0 ± 24.2	5.1 ± 4.0	87.4 ± 28.5	5.3 ± 1.7	9.5 ± 4.7^§^	1.6 ± 0.8^§^	33.7 ± 11.7^§^	2.1 ± 0.7^§^	609.7 ± 22.0¤	1.636 ± 0.059¤	33.7 ± 4.1
3 T (2)	36.6 ± 13.2	4.2 ± 1.5	47.9 ± 17.9	4.1 ± 1.5	27.0 ± 7.0	3.1 ± 0.8	37.1 ± 6.0^$^	3.2 ± 0.5^&^	858.2 ± 3.9^&^	1.165 ± 0.003^&^	35.6 ± 3.5
7 T (2)	38.0 ± 7.0	3.6 ± 0.7	34.0 ± 6.1	3.6 ± 0.6	13.2 ± 4.7	1.2 ± 0.4	10.8 ± 2.3^$^	1.1 ± 0.2^¶^	1063.7 ± 25.0^&^	0.940 ± 0.007^&^	30.2 ± 5.1
3 T (8°)	30.3 ± 19.9	3.1 ± 2.6	45.5 ± 29.2	3.1 ± 2.6	44.3 ± 12.7^1^	5.4 ± 1.6^2^	65.6 ± 13.5	5.2 ± 1.1	814.4 ± 28.0	1.229 ± 0.043	41.5 ± 5.2^Þ^

*Note*: Values for angular features of (1) increasing T_1_ or decreasing R_1_ from 0° to 90° and (2) the broad hump or dig around 40° were analyzed from angular plots in high FA WM shown in Figure 2. “1.5 T (2),” “3 T (2),” and “7 T (2)” refer to data obtained by fitting T_1_ from MP2RAGE scans with four longest TI time images. “3 T (8°)” refers to MP2RAGE images acquired with 8° read flip angle. The 0° degree T_1_ and R_1_ data points at 1.5 T were estimated by linear extrapolation of values from 4.5° to 13.5° (Figure 2A,B). The baseline in T_1_ and R_1_ versus $\theta_{\mathrm{FB}}$ for each microstructural index was taken to be the straight line joining T_1_ or R_1_ points between the means of values from 0° to 20° and 80° to 90°.

Abbreviations: FA, fractional anisotropy; WM, white matter.

* *p* = 0.0025 relative to the 1.5 T value.

^‡^ *p* = 0.0001 relative to the 1.5 T value.

^§^ *p* = 0.0004 relative to the value in the respective data obtained from 6 TI images.

^¤^ *p* = 0.0043 relative to the value in the respective data obtained from 6 TI images.

^&^ *p* = 0.001 relative to the value in the respective data obtained from 6 TI images.

^$^ *p* = 0.0168 relative to the value in the respective data obtained from 6 TI images.

^¶^ *p* = 0.0425 relative to the value in the respective data obtained from 6 TI images. (1) *p* = 0.0226, (2) *p* = 0.0141, Þ *p* = 0.0234, 4° data relative to 8° data, Student's unpaired t‐test. FWHM stands for Full‐Width‐at‐Half‐Maximum and is given in degrees of the fiber‐to‐field angle. Values are mean ± SD. Data are from eight volunteers at 1.5 T and six at 3 T and 7 T.

**FIGURE 3:**
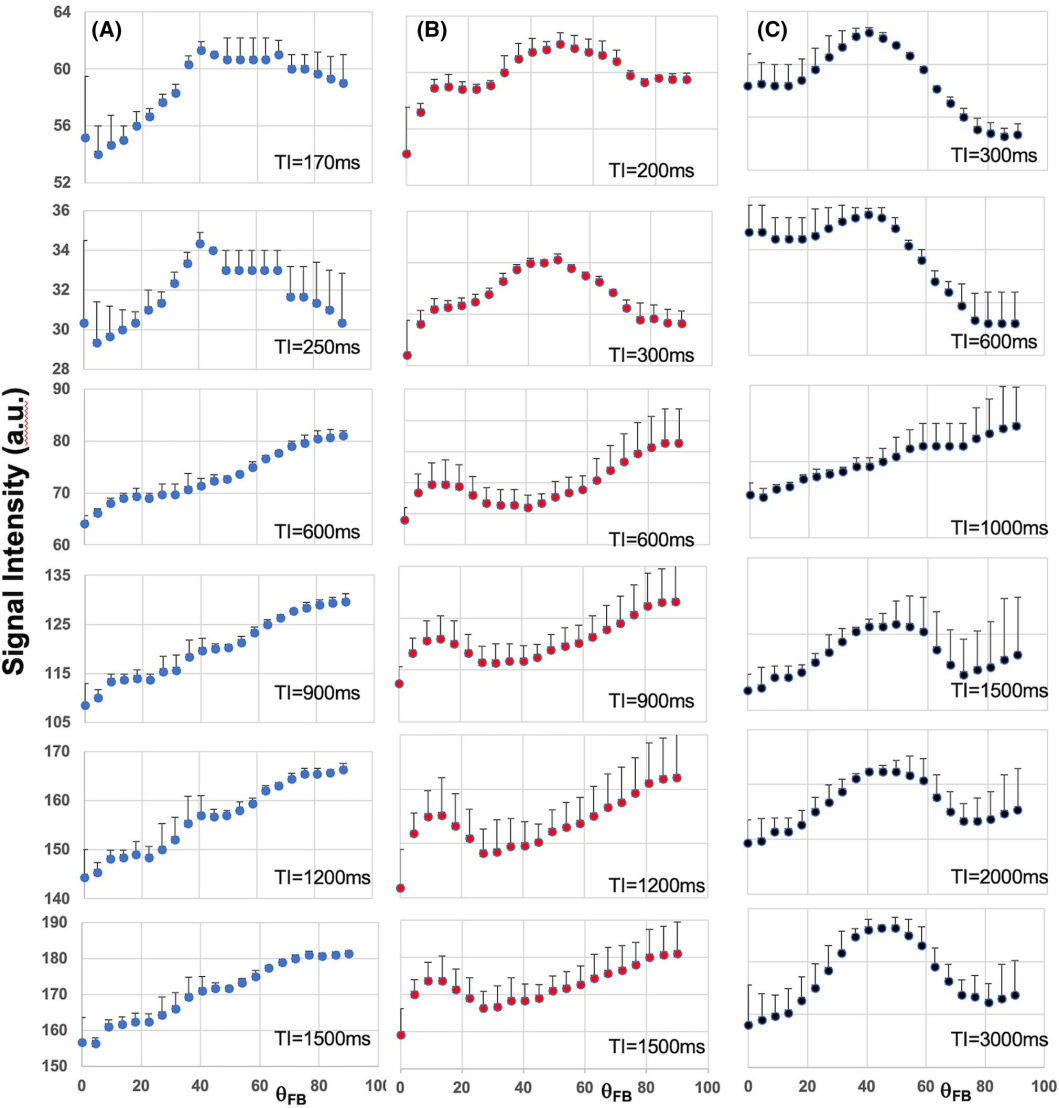
Signal intensities in TI images in high fractional anisotropy (FA) white matter (WM) as a function of fiber‐to‐field angle. (A) Shows the signal intensities of TI images acquired at 1.5 T as a function of θ_FB_. (B) The TI signal data are from 3 T and in (C) from 7 T. TI times are displayed in each subpanel. Blue symbols represent for 1.5 T, red for 3 T, and black for 7 T field strength. Note that signal intensity axes are displayed for reference only for 1.5 T. Data are mean ± SD from eight volunteers at 1.5 T and from six volunteers at 3 T and 7 T.

The signal intensities of each TI time images in high FA WM were plotted as a function of θ_FB_ (Figure 3). At 1.5 T and 3 T the broad humps between 40° and 60° were present in two short TI images (Figure 3A,B,) but absent in longer TI images that showed greater signal at 90° than at 0°. In TI >600 ms images at 3 T, a shallow inverted hump was seen at θ_FB_ ranges of 30° to 60° (Figure 3B). At 7 T, signals in TI images showed a different pattern, that the broad humps between 40° and 50° were present in all TI images (Figure 3C) except that at TI = 1000 ms.

**FIGURE 4:**
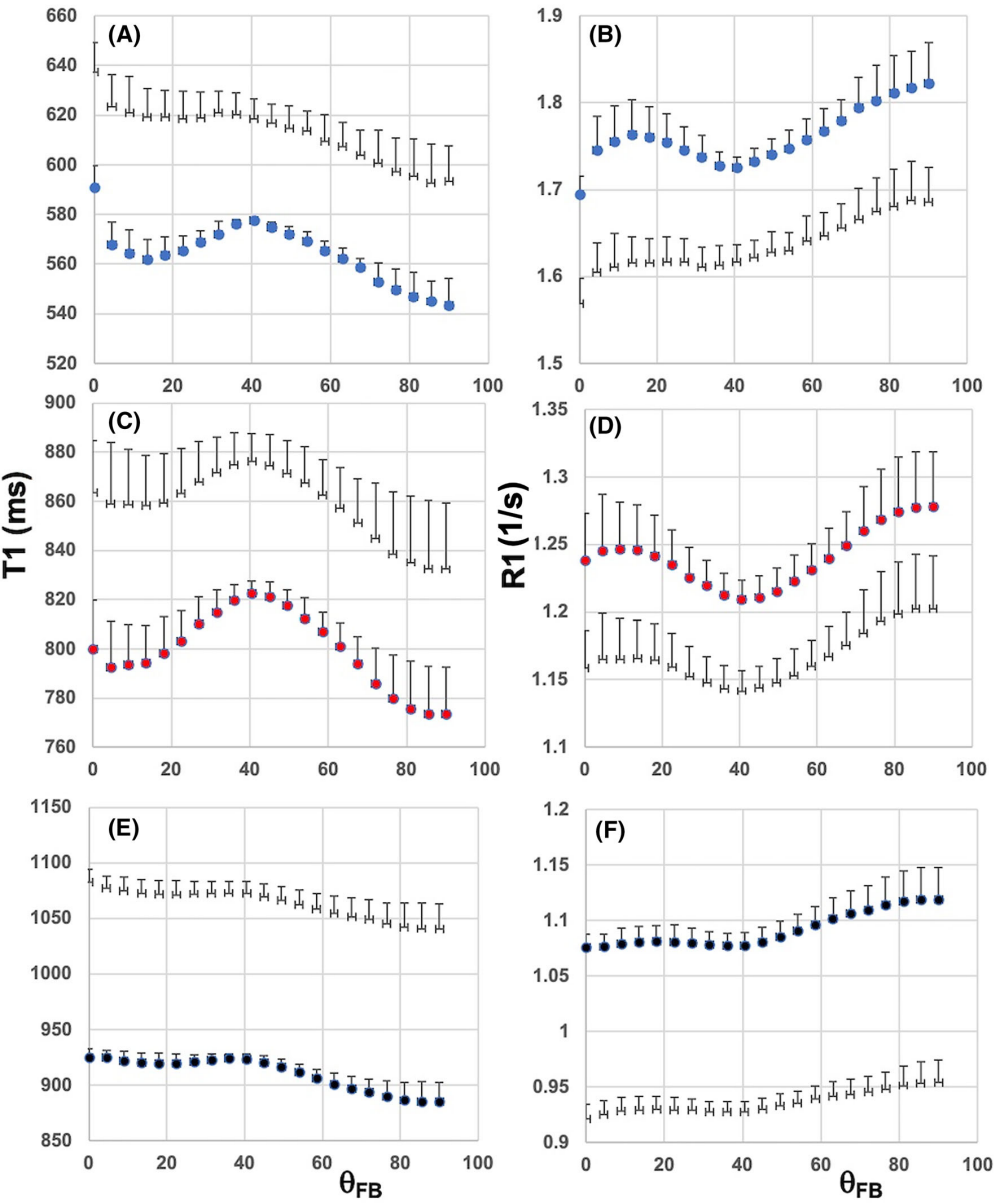
T_1_ and R_1_ as a function of fiber‐to‐field angle in human white matter (WM) at 1.5 T, 3 T and 7 T. (A) Shows T_1_ (in ms) and (B) R_1_ (in 1/s) as a function of θ_FB_ at 1.5 T. (C) Displays T_1_ and (D) R_1_ as a function of θ_FB_ at 3 T. (E) Shows T_1_ and (F) R_1_ as a function of θ_FB_ at 7 T. The blue symbols represent 1.5 T, red 3 T, and black 7 T. The data were obtained from all six TI images, and open symbols in each panel represent longitudinal relaxation data computed from the longest four TI images. Data are from high fractional anisotropy (FA) WM and shown as mean ± SD from eight volunteers in (A,B) and from six volunteers in (C,F).

The findings described above (Figure 3) prompted us to compute T_1_ maps using the four longest TI images only. It is worth keeping in mind a proviso in regard to T_1_ values, because input data of such a fitting has a limited cover of the IR curve and lacks null point and fully recovered magnetization data. The angular patterns of T_1_ and R_1_ values obtained from fitting of all TI and long TI images are shown (Figure 4). T_1_ obtained using the long TI images turned out to be longer than that from all TI images (Figure 4A,C,E), with respectively lower R_1_ (Figure 4B,D,F). The angular plots at 1.5 T of T_1_ from long TI data showed a barely detectable hump around 40° relative to those from all TI images, but the plots had comparable differences in T_1_ and R_1_ between 0° and 90° (Figure 4A,B). The angular plots both at 3 T and 7 T were visually comparable (Figure 4C–F). Quantitative data of the two angular features showed that the 40° humps in the long TI data were greatly reduced relative to all TI data at 1.5 T, as was the difference in R_1_ between parallel and perpendicular tissue (Table 1). The value of the 40° hump relative to the average R_1_ was also smaller in long TI data than that in all TI data at 3 T and 7 T.

Next, we examined angular patterns of longitudinal relaxation measures at 3 T using MP2RAGE data acquired using both 4° and 8° flip read pulses. T_1_ and R_1_ values obtained by the two flip angles were similar, instead, the T_1_ value in the 40° angular feature was greater in images obtained by the 8° flip read pulse than those obtained by 4° (Table 1). FWHM of the broad 40° hump was wider in the former data set than in the latter (Table 1). This was because of increased contributions of angular features at θ_FB_ >45°, a shifting to the right of the broad feature.

**FIGURE 5:**
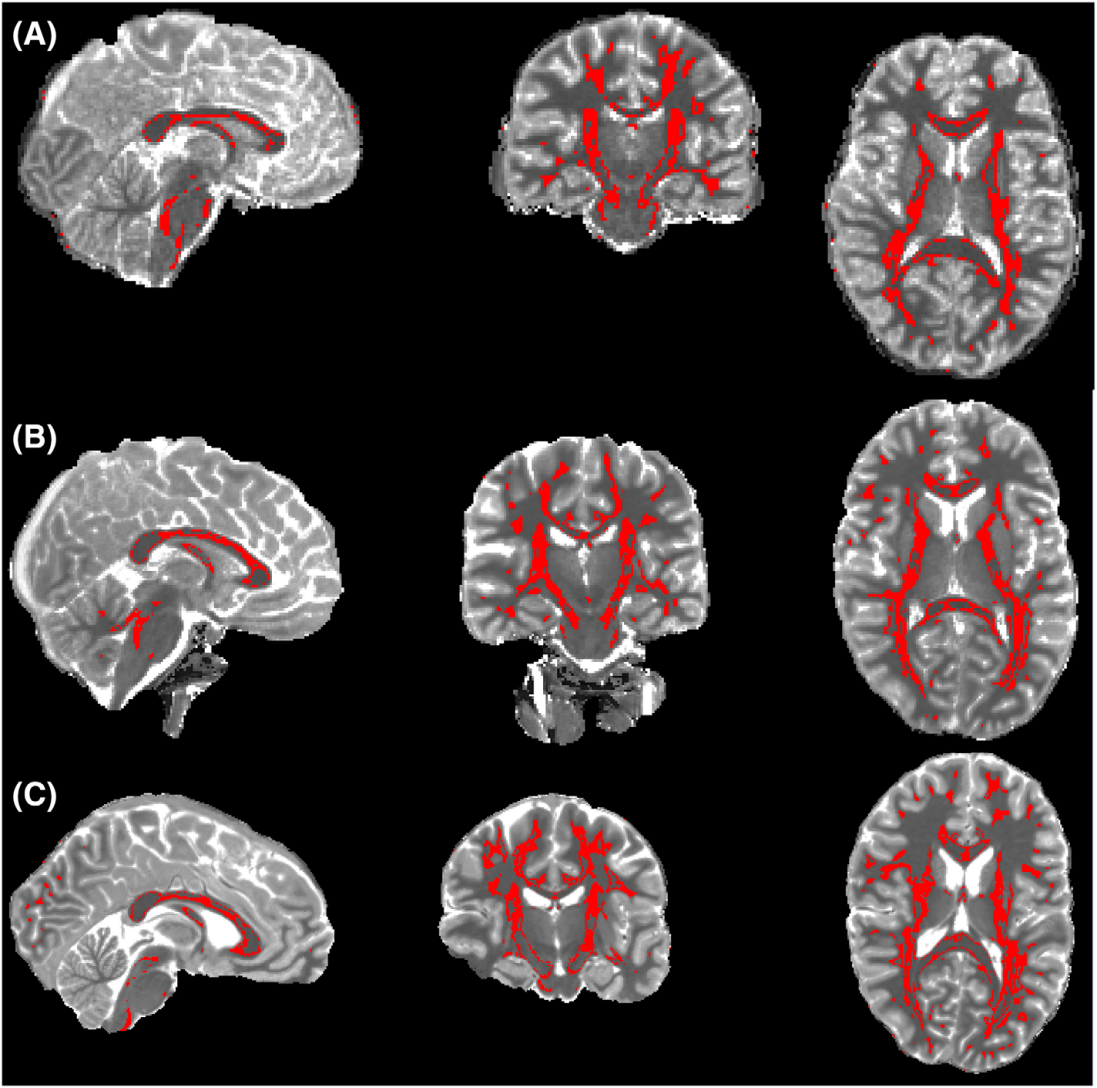
Anatomic distributions of voxels in high fractional anisotropy (FA) white matter (WM) analyzed for T_1_ angular patterns. Representative high FA masks (in red) are overlaid on the T_1_ maps at 1.5 T (A), 3 T (B), and 7 T (C).

The anatomical locations of high FA WM tissue used in T_1_ and R_1_ analyses above are shown in Figure 5. The WM tissue volumes were 148.6 ± 17.4 mL, 199.0 ± 19.8 mL, and 206.0 ± 32.1 mL and 1.5 T, 3 T, and 7 T, respectively. The smaller volume at 1.5 T (*p* = 0.0003 vs. 3 T and *p* = 0.0015 vs. 7 T, unpaired t‐test) was because of lesser contributions from parietal and occipital WM (Figure 5). We downsampled 3 T and 7 T images by twofold to get the spatial resolution of high field images closer to those used at 1.5 T. This resulted in WM volumes of 146.0 ± 26.2 mL (not significant [n.s]. vs. 1.5 T) and 171.8 ± 29.8 mL (n.s. vs. 1.5 T) at 3 T and 7 T, respectively, indicating that lesser WM coverage at 1.5 T was caused by the low spatial resolution relative to those at 3 T and 7 T. The T_1_ versus θ_FB_ plots in downsampled 3 T and 7 T images showed non‐different angular plots to those obtained from high resolution images (Figure S2).

## 4 DISCUSSION

Qualitatively similar T_1_ and R_1_ angular patterns were observed in human WM with high FA at all three field strengths. Relaxation anisotropy results in two angular features in absolute T_1_ images as follows: (1) increasing T_1_ relaxation from parallel to perpendicularly oriented axon tracts and (2) the presence of a broad long T_1_ hump centered around a 40° fiber orientation with respect to B_0_. Quantitative analyses of the two angular features revealed that the Δ T_1_ and Δ R_1_ between 0° and 90° orientations amounted to 3.7% to 4.9% of the average T_1_ and R_1_ in WM at all fields studied. Conversely, the broad hump around 40° showed differing quantitative values so that at 1.5 T and 3 T it was ˜4% of the average T_1_ and R_1_, but only to ˜1.8% at 7 T indicating that the 40° angular feature becomes weaker at the ultra‐high field. It is important to note that the angular patterns of T_1_, as acquired by MP2RAGE MRI, obtained from an ex vivo spinal cord preparation and rotated in a 3 T scanner^23^ show precisely the two features above in agreement with our observation in the human high FA WM in vivo at 3 T (Figure 2). These data demonstrate that the T_1_ relaxation anisotropy is an inherent MR contrast in WM in vivo, and according to the current results, it can be observed at all B_0_s most commonly used in neuroimaging.

The longitudinal relaxation in WM follows a bi‐exponential model consisting of a B_0_‐dependent short component and a B_0_‐independent long component.^10^, ^12^ As stated in the Introduction section, the short T_1_ component arises from immobilized protons in and magnetically coupled protons to those in membranes and myelinated axonal fibers,^8^, ^9^, ^11^, ^44^ whereas the long T_1_ component originates from water protons in intracellular and extracellular spaces. Protons of the long T_1_ component have high rotational freedom so that motional narrowing eliminates residual dipolar couplings, hence, the Lorentzian line shape and isotropic relaxation. The short T_1_ component of immobilized protons in membranes and myelinated axonal fibers experience a structural order resulting in restricted rotational and translational mobility in an inherently anisotropic environment in which one or several components of the Hamiltonian are not averaged out, hence anisotropic relaxation.^26^, ^27^ Sukstanskiy and Yablonskiy recently proposed the so‐called lateral diffusion model (LDM) of immobilized protons bound to macromolecules where restricted lateral diffusion of long lipid molecules modulates dipole–dipole interactions between protons underpinning both anisotropic longitudinal and transverse relaxations.^27^ LDM estimates a unique absorption line shape of immobilized macromolecule‐bound protons because of anisotropic relaxation so that angular patterns of longitudinal relaxation in lipid bilayers and axonal fibers differ. In “cigar shape” axonal fibers, LDM predicts an increasing relaxation from parallel to perpendicular orientation.^27^ Schyboll and co‐workers^26^ proposed an alternative physical explanation for the increasing longitudinal relaxation in perpendicularly oriented axonal tracts relative to those parallel to the field using molecular dynamics simulations. They estimated that the longitudinal relaxation of both solid myelin and myelin‐associated water protons depend on axon fiber orientation and that residual dipolar couplings between hydrogens in an anisotropic molecular environment cause orientation dependency of T_1_, hence, invoking a specific water pool in WM that may be involved in T_1_ anisotropy.^26^ However, neither of the models above dealt with the 40° angular feature. Pampel et al.,^14^ working in the context of the BSB model, estimated that the orientation dependency of MT in WM results from specifically defined dipolar line shapes of a motionally restricted proton pool in the geometric arrangement of lipid bilayers wrapped around axonal fibers. Pampel et al.^14^ also showed that the anisotropic environment in axonal fibers results in orientation dependency of the ultra‐short $T_{2}^{b}$ (of μs range), which show an abrupt prolongation at ˜35° with further long values toward perpendicular orientation. They concluded that the $T_{2}^{b}$ relaxation anisotropy causes orientation dependency of MT, and therefore, the angular feature observed in T_1_ data around ˜40° could correspond to the presence of $T_{2}^{b}$ bias in the quantitative images.^23^ More recently, Yablonskiy and Sukstanskiy^13^ introduced a novel model to physically explain the transfer of anisotropic relaxation of immobilized lipid protons to the bulk water involving quantum hydrogen interactions in a setting of hydrogen‐bond‐driven structural order, the so‐called transient hydrogen bond (THB) model. The THB was tested against the T_1_ values reported from the human brain at a wide range of field strengths showing excellent agreement between the measured and estimated data. The THB model not only predicts a B_0_‐dependency of T_1_, but it also explains the transfer of orientation dependent relaxation of axonal immobilized protons to bulk water. Furthermore, the THB model predicts both the increasing T_1_ relaxation from parallel to perpendicular orientation and the long T_1_ feature at 40°, both of these experimentally observable in WM in vivo^25^ and ex vivo.^23^ The current data show that both angular features, as predicted by the THB model, prevail in WM T_1_ maps at all relevant human neuroimaging field strengths. Our data are also in agreement with a previous study indicating that the effects of multi‐exponential T_1_ in WM are indirectly observable by MP2RAGE T_1_.^45^

It is well known that absolute T_1_ values in vivo vastly differ in data sets acquired with different pulse sequences even when images are collected using the same scanner at a single site.^6^ In fact, an international working group is addressing the issue in a multisite setting to find a common ground for T_1_ relaxometry.^46^ Mindful of these MR technical limitations, the T_1_ images in the current study were acquired with the MP2RAGE sequence incorporating adiabatic inversion pulses and hard readout pulses with the same nominal read flip angles. The MP2RAGE is a time‐efficient IR method for acquiring TI images providing a large anatomical coverage for absolute T_1_ fitting. Initial magnetization immediately following the adiabatic inversion pulse of ˜10 ms in MP2RAGE contains contributions from myelin‐associated and bulk water with minimal (or no) direct contributions from immobilized protons in myelin or non‐myelin macromolecules, although the magnetic interaction of immobilized protons with bulk water protons will contribute to the MP2RAGE images.^7^ Absolute T_1_ values obtained by IR‐based sequences using an adiabatic inversion pulse and TI images ranging from milliseconds to seconds are shorter than those measured with hard inversion pulses and identical TI times in WM ex vivo.^23^ However, the angular patterns in T_1_ images in WM obtained by soft and hard inversion pulses turned out to be quantitatively similar, thereby indicating that relaxation anisotropy does not directly require inversion of non‐aqueous species in an IR‐based MRI.^23^ Instead, both the read flip angle in MP2RAGE (Figure S2) and the use of saturation‐based MRI methods to collect T_1_ images, such as in VFA and multi‐parametric mapping, quantitatively influences the angular T_1_ components.^23^ In our data, T_1_ images acquired with an 8° flip read pulse showed a higher and wider 40° angular feature than in the images acquired with a 4° flip read pulses, yet the overall T_1_ in WM were similar. Further, the size of the 40° angular feature (˜10%) has been shown to be greater in T_1_ images acquired using VFA MRI with higher excitation flip angles than used in the MP2RAGE MRI (˜3%–4%).^23^ The angular patterns of MT saturation and MT ratio have been reported to peak around a 40° orientation in WM.^23^, ^38^ Taking together our in vivo and the ex vivo data by Wallstein and co‐workers,^23^ it can be speculated that “classical MT” contributes to the 40° angular feature. This is interesting in regard to the physical mechanism(s) underpinning T_1_ anisotropy in WM suggesting that orientation‐dependency of $T_{2}^{b}$ “shine through” in T_1_ data acquired using MP2RAGE MRI.^14^

The angular plots of signal intensities in the individual TI images show that the T_1_ relaxation times of the protons in WM microstructure underpinning the relaxation anisotropy are strongly B_0_‐dependent. At 1.5 T TI signals in the θ_FB_ range from 40° to 60° formed a hump‐like appearances only in images with TI <600 ms, whereas at 7 T the hump of TI signals around 40° persisted even in TI = 3000 ms images. Therefore, protons giving the orientation‐dependent T_1_ feature at 1.5 T around 40° have (nearly) fully relaxed before TI = 600 ms, whereas at 7 T they have much longer “life‐time” leading to similar angular patterns in signals of TI images of 300 ms and 3000 ms. Furthermore, the angular patterns in relaxometric images computed without the two shortest TI images showed that the 40° hump was hardly detectable at 1.5 T, but only moderately reduced in size at 3 T and 7 T. Instead, in the “long TI maps of T_1_” the increased T_1_ relaxation from parallel to perpendicular axon was similar in both relaxometric data sets at all three fields. It should be stressed that using only four T_1_‐weighted images without images of passing the null point and fully recovered magnetization, is difficult for T_1_ estimations, and therefore, the T_1_ values should be interpreted with caution. Having said this, it has been reported that the 40° angular T_1_ component in an ex vivo WM preparation was not present in T_1_ plots computed with TIs >450 ms at 3 T, whereas the angular feature of increased T_1_ from parallel to perpendicular orientation remained unaffected.^23^ T_1_ relaxation time of the short component has been estimated in human WM in vivo at a wide range of field strengths from 0.55 T to 7 T.^12^ It was concluded that the short T_1_ component scale exponentially with B_0_.^12^ T_1_ of the short component was estimated to occur at ˜120 ms at 1.5 T, ˜260 ms at 3 T, and ˜540 ms at 7 T.^12^ Providing a single value for the short T_1_ component of WM is not unproblematic because of the complex chemical compositions of myelinated protein microstructures (i.e., axonal fibers) where lipid and protein moieties may have different relaxation dispersions. Nevertheless, the data reported here point to greatly differing T_1_ of the short T_1_ component at 1.5 T relative to 7 T in WM and align the data obtained by IR MRI in WM in vivo.^12^ This fact should be considered when choosing image acquisition parameters at different field strengths, because T_1_ angular patterns are influenced by these in a B_0_‐dependent fashion.

There are some items that should be considered when interpreting the T_1_ results of the current study. It is common to studies where θ_FB_ in vivo are estimated from various microstructural models of diffusivity, such as a diffusion tensor model, ball‐and‐stick model, or fixel‐based model, that only specific volumes of WM can be analyzed for angularly dependent relaxometric measures including T_1_ (see Hutchinson et al.^19^). There are also factors specific to the study that should be considered as follows: first, we used an IR‐based pulse sequence to collect images with relatively long inversion times, the shortest TI was 170 ms at 1.5 T, 200 ms at 3 T, and 300 ms at 7 T. Under these pulsing conditions direct contributions from short T_1_ components are limited, however, on the other hand, fitting IR data to mono‐exponential can be justified. It should also be noted that the choice of the shortest TIs was stipulated by the scanner related restrictions on the spatial resolutions used. Second, spatial resolutions varied between fields both in MP2RAGE and dMRI images. The low spatial resolution of dMRI at 1.5 T may explain the lower anatomical coverage of parietal and occipital WM than incorporated at 3 T and 7 T. Third, the volunteers scanned at 1.5 T were not the same as those examined at 3 T and 7 T. However, as young healthy adults were scanned at all fields, it is expected that WM health and microstructure did not vary among the volunteers. Finally, mostly young volunteers participated in the study, hence, the conclusions about relaxometric and dMRI data are relevant for young adults only.

In conclusion, we have shown that T_1_ relaxation anisotropy is an inherent MRI contrast in WM that can be used in human neuroimaging to complement microstructural data obtained by other MRI techniques. Two angular features of (1) increasing T_1_ relaxation from parallel to perpendicular axonal fiber orientation and (2) a broad long T_1_ hump centered around 40° are present in WM at all field strengths used in human neuroimaging. Our data indicate that the choice of T_1_ data acquisition parameters influences quantitative characteristics of T_1_ angular features particularly at 1.5 T and that magnetic field dependency of the short T_1_ component is a central factor determining the T_1_ angular features. T_1_ anisotropy arising from magnetic interactions between “WM building molecules,”^13^ such as myelination, and bulk water appears to link the MRI contrast to specific components of microstructure, therefore, offering a unique means to image WM microstructure in health and disease. Interestingly, it has been reported that the degree of T_1_ anisotropy decreases with aging^21^ adding it as a potential imaging marker of neurodegeneration.

## Supporting information


**Table S1.** MRI acquisition parameters for diffusion MRI and MP2RAGE.
**Table S2.** Signal‐to‐noise ratios for dMRI images at 1.5 T, 3 T and 7 T.
**Figure S1.** T1 and R1 plotted as a function of fiber‐to‐field angle in human WM with two FA ranges at 1.5 T, 3 T and 7 T.
**Figure S2.** T1 vs. fiber‐to‐field angle in images at two spatial resolutions in high FA WM at 3 T and 7 T.

## Data Availability

The data that support the findings of this study are available from the corresponding author upon reasonable request.
