# Supplementary material for: Fiber orientation‐dependent T1 angular features in human white matter at 1.5 T, 3 T, and 7 T
Source: Magn Reson Med. 2025 Jul 25;94(6):2611–23. doi: 10.1002/mrm.70009 (PMC12501718; doi:10.1002/mrm.70009)
Supplement: Supplementary file 1 — Table S1. MRI acquisition parameters for diffusion MRI and MP2RAGE. Table S2. Signal‐to‐noise ratios for dMRI images at 1.5 T, 3 T and 7 T. Figure S1. T1 and R1 plotted as a function of fiber‐to‐field angle in human WM with two FA ranges at 1.5 T, 3 T and 7 T. Figure S2. T1 vs. fiber‐to‐field angle in images at two spatial resolutions in high FA WM at 3 T and 7 T. [file MRM-94-2611-s001.docx]

**SUPPLEMENTARY MATERIAL**

**FIBRE ORIENTATION-DEPENDENT T1 ANGULAR FEATURES IN HUMAN WHITE MATTER AT 1.5T, 3T AND 7T.**

Risto A. Kauppinen^1^, Ekaterina Paasonen^2,3^, Jeromy Thotland^4^, Mervi Könönen^5^, Pramod Pisharady^4^, Christophe Lenglet^4^, Juhana M. Hakumäki^5^, Olli H.J. Gröhn^2^ and Michael Garwood^4^

^1^Department of Electric, Electronic and Mechanical Engineering, University of Bristol, Bristol, UK. ^2^A.I.Virtanen Institute, University of Eastern Finland, Kuopio, Finland; ^3^Kuopio University Hospital Neurocenter, Kuopio, Finland. ^4^Center for Magnetic Resonance Research, University of Minnesota, Minneapolis, MN, USA and ^5^Department of Radiology, University Hospital of Kuopio, Kuopio, Finland.

**Table S1.** MRI acquisition parameters for diffusion MRI and MP2RAGE

| **PARAMETER** | **1.5T dMRI** | **3T dMRI** | **7T dMRI** | **1.5T MP2RAGE** | **3T MP2RAGE** | **7T MP2RAGE** |
| --- | --- | --- | --- | --- | --- | --- |
| Voxel size (mm) | 2.0x2.0x2.0 | 1.5x1.5x1.5 | 1.05x1.05x1.05 | 1.6x1.6x1.6 | 1.2x1.2x1.2 | 0.9x0.9x0.9 |
| Slices | 72 | 92 | 128 | 3D | 3D | 3D |
| TR (ms) | 5500 | 3230 | 7000 | 1650 | 2000 | 3540 |
| TE (ms) | 84.0 | 89.2 | 71.2 | 1.17 | 1.68 | 1.34 |
| MP2RAGE readout pulse and duration | - | - | - | 4^o^ rectangular pulse, 99 μs | 4^o^ slab-selective sinc pulse, 1,000 μs | 4^o^ rectangular pulse, 100 μs |
| GRAPPA | - | - | 3 | 2 | 3 | 3 |
| Phase PF | - | - | - | - | 6/8 | 6/8 |
| Slice PF | - | - | - | - | 6/8 | 6/8 |
| Phase encoding | A>>P, P>>A | A>>P, P>>A | A>>P, P>>A | Linear | Linear | Linear |
| Gradient directions | 20 (AP), 20 (PA) | 197(AP), 197 (PA) | 143(AP), 143(PA) | - | - | - |
| b-values (s/mm^2^) | 750, 1500 | 1500.3000 | 1000,2000 | - | - | - |
| b=0 s/mm^2^ volumes | 2(AP), 2(PA) | 13(AP), 17(PA) | 11(AP), 13(PA) | - | - | - |
| TI (ms) | - | - | - | 170, 250, 600  900, 1200, 1500 | 200,300,600  900,1200,1500 | 300, 600,1000  1500,2000, 3000 |
| Scan duration (min:sec) | 12:16 | 22:38 | 30:48 | 6:06 | 7:45 | 14:03 |

‘PF’ stands for partial Fourier,

**Table S2.** Signal-to-noise ratios for dMRI images at 1.5T, 3T and 7T

| **B0/ b-value** | **b=0 SNR** | **X SNR** | **Y SNR** | **Z SNR** |
| --- | --- | --- | --- | --- |
| 1.5T/ 750 | - | 9.2±2.3 | 22.0±5.2 | 20.9±4.9 |
| 1.5T/ 1500 | 30.7±7.3 | 4.6±1.0 | 17.9±4.2 | 16.5±3.8 |
| 3T/ 1500 | 16.6±7.1 | 3.1±1.2 | 9.2±4.0 | 9.4±4.0 |
| 3T/ 3000 | 18.2±7.8 | 2.2±0.8 | 7.7±3.3 | 7.9±3.2 |
| 7T/ 1000 | 10.2±3.8 | 3.2±1.2 | 6.4±2.4 | 6.5±2.4 |
| 7T/ 2000 | 10.2±3.8 | 3.2±1.2 | 6.4±2.4 | 6.5±2.4 |

SNRs as determined by DIPY procedure (see Methods section) are given for b=0 s/mm^2^ and each principal gradient axis at 1.5T with b-values 750 s/mm^2^ and 1500 s/mm^2^ , at 3T scans acquired with b-values of 1500 s/mm^2^ and 3000 s/mm^2^ and for 7T with b-values of 1000 s/mm^2^ and 2000 s/mm^2^. Values are mean ± SD for 8 volunteers at 1.5T, and 6 volunteers at 3T and 7T.


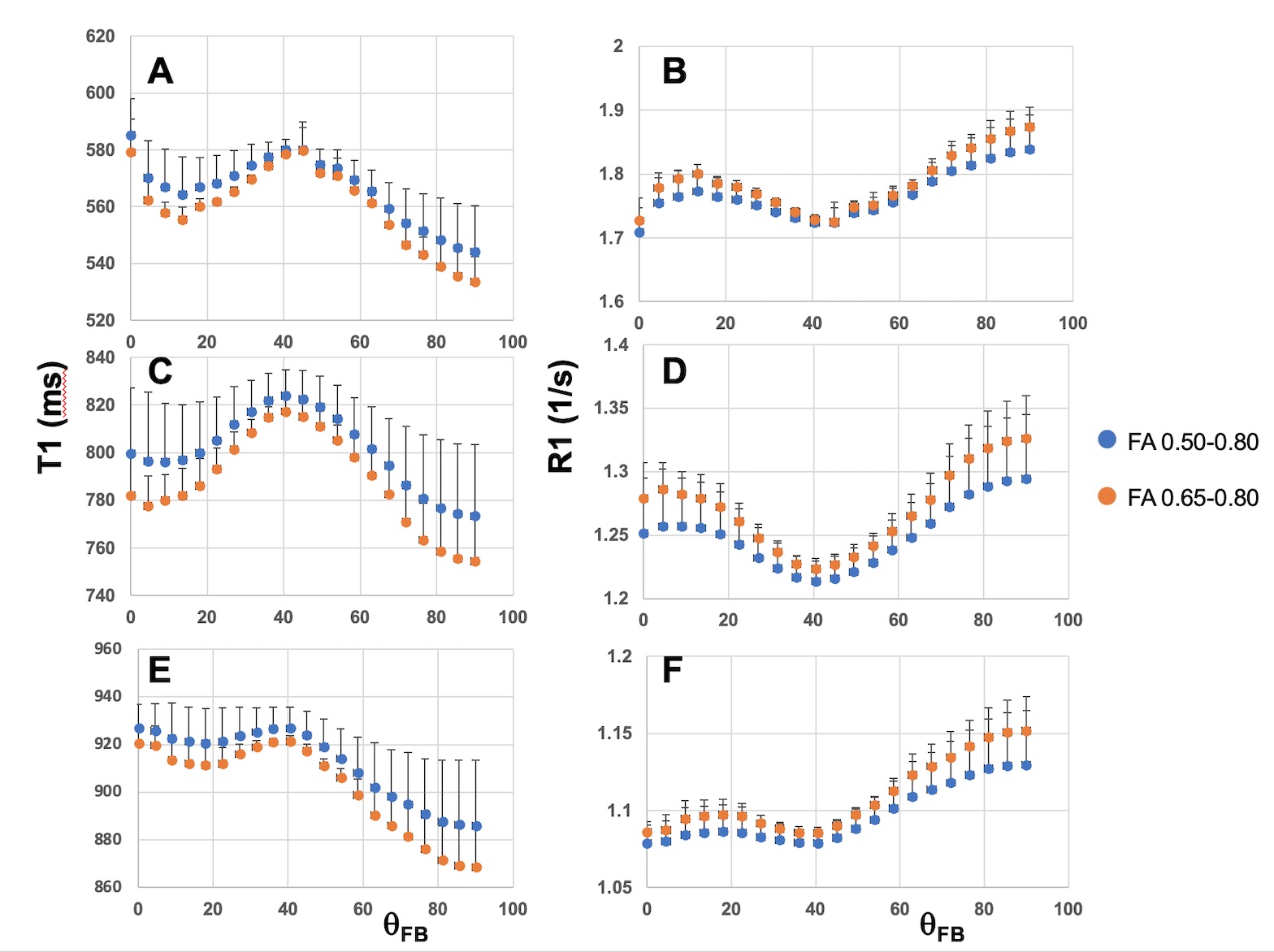


**Figure S1.** T1 and R1 plotted as a function of fibre-to-field angle (θ_FB_) in human WM with two FA ranges at 1.5T, 3T and 7T.

1D T1 (in ms) and R1 (in 1/s) as a function of θ_FB_ plots are shown from 1.5T (A and B), 3T (C and D) and 7T (E and F) from WM with two FA ranges, blue symbols for the FA range from 0.50 to 0.80 (range 1) and orange symbols from FA 0.65 to 0.80 (range 2). The T1 values were 566.3±15.0 ms for the range 1 and 560.3±14.7 ms for the range 2 at 1.5T, the respective values were 801.0±24.8 ms and 788.0±22.0 ms at 3T and 912.0±21.3 ms and 902.1±20.3 ms at 7T. R1 values for the range 1 were 1.767±0.047 1/s and for the range 2 1.786±0.048 1/s at 1.5T. The respective R1 values were 1.250±0.039 1/s and 1.270±0.036 1/s at 3T and 1.097±0.026 1/s and 1.109±0.025 1/s at 7T. The volumes in the range 2 WM were 54.1±8.8 ml, 48.5±8.2 ml and 54.9±10.3 ml at 1.5T, 3T and 7T, respectively. The values are mean ± SD from 8 volunteers at 1.5 and from 6 volunteers at 3T and 7T.


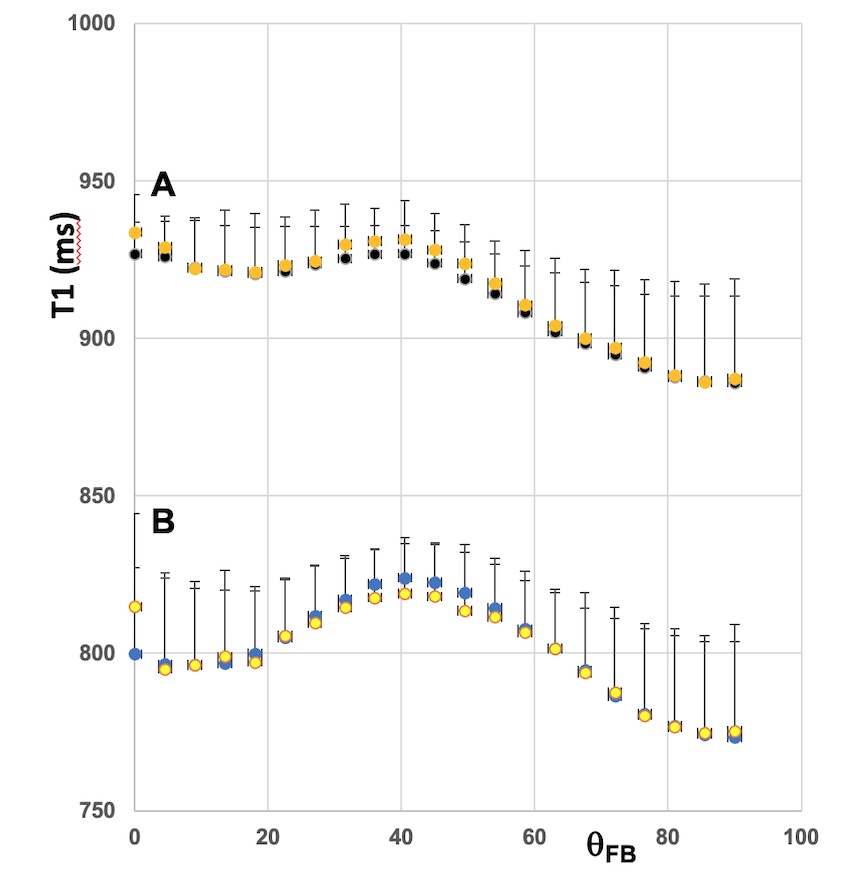


**Figure S2.** T1 vs fibre-to-field angle plots in images at two spatial resolutions in high FA WM at 3T and 7T.

MP2RAGE images were down-sampled two-fold, DTI images registered to the lowered resolution and 2D T1 (in ms) vs fibre-to-field plots analysed in Matlab. In panel (A) data from high FA WM are shown by black symbols at 0.9 mm^3^ resolution and yellow symbols at 1.8 mm^3^ resolution images at 7T. In (B) the blue symbols show values at 1.25 mm^3^ resolution and yellow at 2.5 mm^3^ resolution at 3T. Data are shown as means ± SD from six volunteers at both fields.
